# Non-technical skills of anaesthesia providers in Rwanda: an ethnography

**DOI:** 10.11604/pamj.2014.19.97.5205

**Published:** 2014-09-26

**Authors:** Patricia Livingston, Lauren Zolpys, Christian Mukwesi, Theogene Twagirumugabe, Sara Whynot, Anna MacLeod

**Affiliations:** 1 Department of Anesthesia, Pain Management and Perioperative Medicine, Dalhousie University, Halifax, Nova Scotia, Canada; 2 Department of Anaesthesia and Intensive Care Medicine, University of Rwanda, Huye Campus, Butare, Rwanda; 3 Division of Medical Education, Dalhousie University, Halifax, Nova Scotia, Canada

**Keywords:** Anaesthesia, non-technical skills, genocide

## Abstract

**Introduction:**

Patient safety depends on excellent practice of anaesthetists’ non-technical skills (ANTS). The ANTS framework has been validated in developed countries but there is no literature on the practice of ANTS in low-income countries. This study examines ANTS in this unexplored context.

**Methods:**

This qualitative ethnographic study used observations of Rwandan anaesthesia providers and in-depth interviews with both North American and Rwandan anaesthesia providers to understand practice of ANTS in Rwanda.

**Results:**

Communication is central to the practice of ANTS. Cultural factors in Rwanda, such as lack of assertiveness and discomfort taking leadership, and the strains of working in a resource-limited environment hinder the unfettered and focused communication needed for excellent anaesthesia practice.

**Conclusion:**

Despite the challenges, anaesthesia providers are able to coordinate activities when good communication is actively encouraged. Future teaching interventions should address leadership and communication skills through encouraging both role definition and speaking up for patient safety.

## Introduction

Patient safety in the operating room is greatly affected by human factors, such as team working, leadership and communication [1]. Estimates are 70 to 80% of anaesthetic and surgical untoward events are caused by errors in human factors [1, 2]. The Anaesthetists’ Non-Technical Skills (ANTS) framework describes human factors relevant to the safe practice of anaesthesia [3]. The ANTS system has four categories: task management, team working, situation awareness and decision-making [3]. Although ANTS have been studied in European, Australian, and North American centres [4–6] there are no reports of this framework in low-income countries. Work in operating theatres in low-income countries is often clinically stressful; resources and role models are scarce [7]. Our study sought to understand the practice of ANTS in this unexplored context.

Rwanda is an East African country where the genocide of 1994 killed nearly one million people. Health professionals were not spared; there was only one anaesthesiologist in the country for years following the genocide [8]. Despite a remarkable regeneration, Rwanda has only 12 consultant anaesthetists for a population of 12 million people [9]. In 2006 the Canadian Anesthesiologists’ Society International Education Foundation initiated a partnership to build anaesthesia capacity at the National University of Rwanda [10]. Based on well-established relationships from the partnership, Rwanda was chosen as an appropriate location to explore the practice of ANTS in a low-income country.

Rwanda is striving to improve human resources but anaesthesia leaders are scarce. Technicians, who have completed a three-year diploma in anaesthesia following high school, provide the majority of anaesthetic services in Rwanda. Consultant anaesthetists work only in two urban centres at teaching hospitals. Anaesthesia services in district hospitals are provided entirely by technicians, who must manage complex patient problems without supervision [11].

Anaesthesia working conditions are difficult. Patients frequently present with advanced pathology, equipment and monitors are inadequate and, at times, the entire country runs out of essential drugs such as muscle relaxants, ketamine or bupivacaine. Thus, the majority of anaesthesia providers must routinely cope with limited supervision, high workload, complex pathology, and insufficient resources. Operating theatres at the two main teaching hospitals are full of trainees in nursing, medicine, anaesthesia technology, and post-graduate specialties. A typical anaesthesia team in a teaching hospital is composed of two anaesthesia providers – either two technicians or a technician and a resident. A supervisor is meant to be in a nearby theatre or available by phone. In addition to the anaesthesia technicians there are many students of either medicine or anaesthesia technology who are learning mainly by observation. Therefore, teaching hospitals in Rwanda have mainly technician anaesthesia providers and many learners, but few experienced supervisors.

Non-technical skills are considered vital to patient safety, yet they remain hard to practice well, even in ideal circumstances. Given the widespread difficulties facing anaesthesia providers in Rwanda, we designed this study to understand how ANTS are practiced in this challenging setting with an intention that knowledge gained would inform subsequent ANTS teaching interventions in the low-resource context.

## Methods

### Ethical considerations

Research Ethics Board approval was obtained from the National University of Rwanda and Dalhousie University prior to undertaking this ethnographic study. Participants were assured their data and comments would be confidential and any direct quotations would be de-identified.

### Study population

Study participants included Rwandan and North American anaesthetists and anaesthesia trainees and Rwandan anaesthesia technicians. We purposively selected a range of participants with diverse experiences to ensure data from a variety of sources (Table 1). Consistent with ethnography, purposive sampling involves finding participants and situations that best help the researchers obtain a rich understanding of the situation being studied [12]. After consent from participants, we conducted in-depth interviews with Rwandan and North American anaesthesia providers and organized focused observations of anaesthesia practice in Rwandan operating rooms.

**Table 1:** Demographic characteristics of participants from whom interview and observation data was obtained

Type of Anaesthesia Provider	Number of Interviews (n=20)	Country of origin		Years of Anaesthesia Experience		
		**Canada/USA**	**Rwanda**	**<5**	**5-15**	**>15**
Technician	3	0	3	1	1	1
Trainee	7	4	3	7	0	0
Anaesthetist	10	6	4	2	3	5
**Type of Anaesthesia Provider**	**Number of Observations (n=10)**			**Years of Anaesthesia Experience**		
				**<5**	**5-15**	**>15**
Technician	4			1	3	
Trainee	5			5		
Anaesthetist	1			1		

### Data collection

#### Interviews

The interview team (CM, LZ, and PL) conducted twenty interviews; ten with Rwandan anaesthesia providers and ten with North American anaesthetists or trainees who had teaching experience in Rwanda. North American interviews were in English and Rwandan interviews were in the participants’ preferred language (English, French or Kinyarwanda) to allow free expression of ideas. Interviews conducted in French were translated into English for data analysis by two of the co-investigators (LZ, CM). Kinyarwanda was immediately translated into English at the time of interview by a Rwandan co-investigator (CM). Prior to the interviews, participants were asked to reflect on situations they had encountered in the operating room where either untoward event had occurred or had the potential to occur but had been prevented. Early in the process of interviewing, we used open-ended interviews asking participants to describe these clinical situations. After a few interviews were completed, emergent themes (e.g. role definition, resignation to poor outcomes) recurred and, therefore, created a semi-structured question guide designed to clarify these themes (Table 2). These questions guided the interview but participants still had opportunity for open-ended discussion. Interviews ranged in length from thirty minutes to one hour. Interviews were audio-recorded then transcribed verbatim by members of the research team (LZ, PL, and SW). Interviewers also kept handwritten notes of the interviews. As we worked with the transcripts and notes, themes emerged from the data. We then used subsequent interviews to clarify and refine data interpretation.

**Table 2:** Question format used to guide interviews with anesthesia providers describing and clarifying the current practice of ANTS in two teaching hospitals in Rwanda

What prevents an anaesthesia provider from recognizing the significance or severity of a situation?
Do you believe that working in a resource poor setting can lead to acceptance of poor outcomes? How so?
How does the presence of multiple anesthesia providers affect ultimate responsibility for anaesthesia care?
If there are multiple anaesthesia providers, do they communicate to coordinate plans? If not, why not? Are individual roles clearly defined?
Do anaesthesia providers and surgeons communicate to coordinate patient care? Examples?
Have you been in a situation where you were uncomfortable bringing up a patient safety concern?
What is the relationship between anaesthesia technicians and anaesthesia residents?
Who is the leader of the anaesthesia care team? Is leadership important?
Is communicating with patients important?
Is anaesthesia care improving in Rwanda? Why or why not?

#### Observations

The Rwandan co-investigators (CM, TT) conducted ten observations of Rwandan anaesthesia providers preparing for and inducing anaesthesia for elective surgery. The observations occurred at two tertiary care teaching hospitals: University Teaching Hospital of Kigali (UTHK) and University Teaching Hospital of Butare (UTHB). Observations focused on recording the everyday events, activities, speech, interactions and behavior of the anaesthesia providers. Consistent with best practice in ethnography, the researchers wrote field notes during the observation period [13]. As we did not want to presume that the ANTS framework was necessarily applicable in this context, we simply described what was observed rather than trying to fit observations to the ANTS framework. As we engaged in iterative and concurrent data collection and analysis, it quickly became apparent that behaviors we observed were congruent with the ANTS framework.

### Data Analysis

Members of the research team (PL, LZ, CM, and SW) used hybrid discourse analysis, informed by Carabine's 11-step process [14], to evaluate raw data from both interviews and observations. Data analysis involved revisiting both the data and emergent concepts until an agreed-upon set of themes was recognized. Themes (e.g. lack of assertiveness) were identified and subsequently clustered into major themes (e.g. culture). Data collection and analysis occurred in an iterative fashion rather than the traditional sequential approach, so that emerging themes informed subsequent data collection. This meant, for example, that the interview style changed from an open-ended approach, where participants described cases, to a semi-structured format in order to clarify themes. In any ethnographic study the investigators are immersed within the situation they are examining. Reflexivity in qualitative research is a process by which researchers account for that immersion, recognizing that all experiences are seen through one's own cultural lens [15]. We approached this study reflexively, in terms of data collection and analysis, by including Rwandan and Canadian anaesthetists, a research facilitator and a qualitative research specialist. This brought diverse perspectives to the study.

## Results

**Figure 1:**
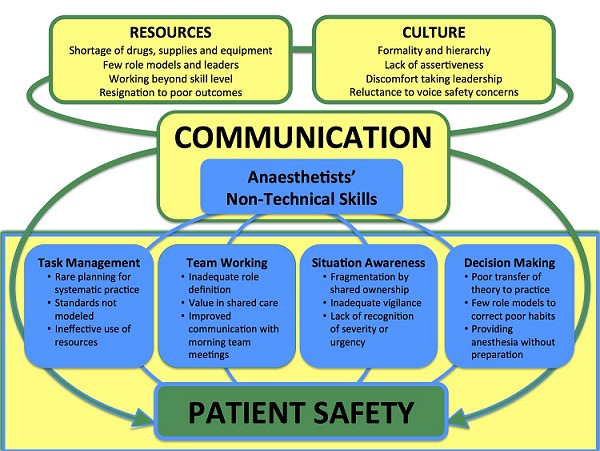
Illustration of the dynamic influences on ANTS in Rwanda. Communication is placed centrally as it influences and pervades the categories of ANTS. Cultural elements (e.g. discomfort taking leadership) and habituation to resource shortages impair effective team communication and coordination. The ANTS domains influence and are influenced by communication patterns (e.g. inadequate role definition). As practice of ANTS affects patient safety, our findings suggest that communication skills should be actively targeted for future teaching interventions to improve ANTS

Communication patterns, central to the practice of non-technical skills, are influenced by two main factors: habituation to working in a resource poor setting and the formal hierarchical culture of operating room personnel in Rwanda. The dynamic interaction between influences on communication and practice of ANTS is summarized in Figure 1.

### Communication

According to the ANTS framework, communication pervades all categories and is not a separate category or element [16]. Poor communication patterns were identified to have a profound effect on ANTS in Rwandan operating theatres. The presence of multiple anaesthesia providers in each operating theatre often led to inadequate communication among members of the anaesthesia team. This was described as:“… Sharing of responsibility but without any definition of responsibility.” (Rwandan anaesthetist). North American anaesthesia study participants attributed the lack of role definition to habitual patterns amongst the anaesthesia team. The Rwandan participants, however, identified poor team coordination to be a result of lack of communication skills and fear of speaking up in a traditionally hierarchical culture. Hierarchy is natural in any organization but there is increasing recognition of the need for “horizontal communication” [17–19]. Horizontal communication within teams who collaborate for patient care improves safety by “creating a culture that empowers subordinates to speak up and encourages senior members to listen” [20]. Interviews and observations in Rwanda showed communication is mostly “top down” and team members who see themselves lower in the hierarchy are reluctant to speak up.

### Resources

Operating theatres in Rwanda have shortages not only of material resources (e.g. drugs, supplies and equipment) but also of human resources (e.g. role models and leaders). Anaesthesia providers frequently find themselves in situations where they must work beyond their skill level without adequate supervision. In such strained circumstances, anaesthesia providers can become resigned to sub-optimal outcomes. “People, at the end of the day, feel resigned. They can't do anything. Someone is coming in a bad situation and is sick. The heart rate is 150 and maybe the patient is old and dehydrated. He receives fluids and is still tachycardic. Sometimes you find an anesthesia provider that says, “Okay. I can't do anything”. (Rwandan trainee). The anaesthesia providers we interviewed expressed frustration with resource limitations and spoke of the desire to improve their ability to care for patients. “We are uncomfortable and concerned, even though we can't do anything about the issue.” (Rwandan technician). “(Rwandan anaesthetist) had his hands full with some other work … and he was trying to call the only guy on his cell phone … who could come and replace the oxygen tanks, … he kept getting his voicemail, and … it was really disastrous for the entire operating room.” (Canadian anaesthetist). “What you have to do is save people's lives, so when the outcome is not good you can be frustrated.” (Rwandan technician).

### Culture

Anaesthesia providers at tertiary care hospitals in Rwanda work in a culture that values formality, politeness and hierarchy. These cultural qualities lead to lack of assertiveness, discomfort taking leadership and reluctance to voice safety concerns. Hierarchical communication barriers are particularly evident between surgeons and anaesthesia technicians. “About 70% of the anaesthesia team would not be comfortable saying something to the surgeon even if it's unsafe.” (Rwandan technician). “A technician sees a senior resident doing something and doesn't want to challenge it. He thinks he doesn't have enough knowledge to give advice to his seniors.” (Rwandan technician).

### Task Management (Observation, UTHK)

A patient came to the OR for debridement of Fournier's gangrene under spinal anaesthesia. The anaesthesia machine in the room was not prepared or checked in anticipation of potential need for general anaesthesia or emergency resuscitation; the oxygen source was not connected to the anaesthetic machine, and there was no laryngoscope, mask, or endotracheal tube in the room. The blood pressure cuff was not functional however this was not determined until after induction of anaesthesia as no pre operative blood pressure was measured.

Task management is defined as “Skills for organizing resources and required activities to achieve goals, be they individual case plans or longer term scheduling issues. It has four skill elements: planning and preparing; prioritizing; providing and maintaining standards; identifying and utilizing resources” [21]. Organizing resources and activities is difficult in a setting of limited materials, high clinical acuity and poor team coordination. Excellent safety standards are hard to maintain with few experienced supervisors. In Rwanda, planning and preparing for anaesthesia is rarely systematic so that relatively simple cases become complex. Existing materials are not employed fully. For example, when the automatic blood pressure machine fails, rather than using a manual sphygmomanometer, the anaesthesia provider may not monitor the blood pressure. Observations frequently revealed lack of preparation of emergency drugs and equipment even when such materials were available.

The (trainee) went ahead and started giving ketamine…and at this point we didn't have suction hooked up, we didn't have the right sized endotracheal tube in the room, the patient was still not positioned appropriately, the headboard was still on the bed, the assistant wasn't applying cricoid pressure and the patient hadn't been pre-oxygenated.” (Canadian trainee). Systematic organization for effective task management has improved with education. “The effort to try and be organized, to organize the equipment, is more in those residents that have been to Canada. They really try to get their stuff together and they are aware that we need this equipment. They wouldn't consider doing a block without monitoring, for example.” (Canadian anaesthetist).

### Team Working (Observation, UTHB)

In one case, we observed a technician giving a spinal anaesthetic to an eleven year old boy with a five-day old leg fracture, and the surgeons started to mobilize the leg without asking if the spinal was done or not. He saw an open wound in the popliteal region and said to the anaesthesia team that he was not going to operate because of that. He left the OR without providing an explanation to the patient or the family. He came back and spoke with the anaesthesia supervisor who asked why they had anesthetized the patient for nothing. The surgeon replied (laughing): “It is not for nothing, we did the mobilization of the knee”. Then the anaesthetist asked: “Now, who is going to pay the bill of that anaesthesia?” The surgeon replied: “The surgical ward nurse who did not comment on the wound before the patient was sent to the OR”.

Team working is defined as “Skills for working in a group context, in any role, to ensure effective joint task completion and team member satisfaction; the focus is particularly on the team rather than the task. It has five skill elements: coordinating activities with team members; exchanging information; using authority and assertiveness; assessing capabilities; supporting others” [21]. Effective team performance under a high workload is dependent on a shared mental model, which is the ability to apply a common understanding of a task by team members [22]. Inadequate role definition by Rwandan anaesthesia providers creates fragmentation of care, whereby several people give anaesthesia with no clarity about who is ultimately responsible for the patient.

I think that the way we do things with three or four people around the head is not a good way because it affects negatively the anaesthesia. There is no leader in the group. You think your colleague gave something but he didn't. It's not good because there's disorder.” (Rwandan trainee). “There was no handover and there would not even be anyone saying to me when they were going. I would just turn around and I would suddenly be giving the anaesthetic because there would be no other anaesthesia provider in the room.” (Canadian trainee).

Rwandan anaesthesia providers, particularly technicians, found value in having two anaesthesia providers work together to share the burden of anaesthesia care in a challenging environment. They explained that having more than two could lead to chaos and poor team coordination. “If you are two people you can prevent leaving the patient alone. You can exchange ideas about the plan. There are many advantages.” (Rwandan technician). Anaesthesia morning team meetings (morning report), implemented over the last few years, have improved coordination of team activities. “Morning report, it's excellent. We should be doing it here in Canada. You know, if I did that here for five years, I'd be a much better clinician… to listen to cases and think about how you would do each of them is very valuable.” (Canadian trainee).

### Situation Awareness (Observation, UTHK)

A two-year-old girl was brought to the operating room for removal of an esophageal foreign body. She was induced under anaesthesia and intubated, and the ETT position was confirmed via auscultation. An anaesthesia resident and technician were both involved in the case, and were busy helping each other secure the tube. Neither of them was ventilating the patient. During this process, they did not recognize that the child's oxygen saturation had fallen to 43%, and only noticed when they were made aware by the surgeon who happened to be in the room.

Situation awareness is defined as “Skills for developing and maintaining an overall awareness of the work setting based on observing all relevant aspects of the theatre environment (patient, team, time, displays, equipment); understanding what they mean, and thinking ahead to what could happen next. It has three skills elements: gathering information; recognizing and understanding; anticipating” [21]. Situation awareness is hindered by the fragmentation of responsibility that comes with shared ownership of the patient. Multiple anaesthesia providers with unclear role definition lead to poor vigilance.

Having multiple providers in the room is not good for the patient … No one is responsible. No one is vigilant. One is at the head and one is giving meds but no one is responsible. Someone thinks someone else is doing something. When there are three, no one is responsible. It is divided.” (Rwandan technician). Inadequate vigilance also stems from lack of knowledge. “If I compare with other countries, like Western countries, vigilance is less but it is a consequence of lack of knowledge.” (Rwandan anaesthetist). “Lack of knowledge. When you don't watch globally the patient. The blood pressure might not drop immediately. It might take time. If the patient is bleeding and you don't recognize it can take time for the blood pressure to drop and you might not recognize until it is too late.” (Rwandan technician). Without recognition of clinical severity, there is often no sense of urgency when decisive action might be required. (Describing an intra-operative oxygen failure and without back up ventilator) “No one else seemed overly concerned about this but my head was exploding.” (Canadian trainee).

### Decision-Making (Observation, UTHK)

A spinal anaesthetic was given to a patient having a penile amputation but failed to provide sufficient anaesthesia for surgery. There were a lot of anaesthesia providers in the room but none of them seemed to consider or suggest other options. They were somehow stuck… The patient was in pain each time the surgeon tried to operate. It was Friday, the surgeon was in a rush to do his case and the block was not working. It wasn't until (Rwandan anaesthetist) entered the room that a solution was identified. He suggested that the surgeon infiltrate with lidocaine, and the operation was able to proceed successfully.

Decision-making is defined as “Skills for reaching a judgment to select a course of action or make a diagnosis about a situation, in both normal conditions and in time-pressured crisis situations. It has three skill elements: identifying options; balancing risks and selecting options; re-evaluating” [21]. With few role models to correct poor habits, good theoretical knowledge is often not integrated into clinical practice. Anaesthesia providers can develop practice patterns without re-evaluating. “Often those theoretical discussions didn't translate into practice because people sort of do things the way they've always done them.” (Canadian anaesthetist). “We talked earlier about theory and practice…not being able to translate the theory to practice, to theoretically understand it's wrong but when confronted with it practically to have trouble.” (Canadian trainee).

A frequent theme we found in both the interviews and observations is that anaesthesia providers often decide to proceed with anaesthesia without adequate evaluation, preparation, equipment, and drugs. Study participants consistently identified consultation with colleagues during morning anaesthesia team meetings as improving decision-making. “At morning report the whole team identified it as a challenging case and sought, I guess, opinions from each other as to what were the anaesthesia considerations and then the best way to manage the case.” (Canadian trainee).

### Improvement

Despite the many barriers to safe anaesthesia care, participants identified improvements through better education and increasing number of staff. “Anaesthesia care has been improving … fifteen years ago there was only one anaesthesia doctor and maybe two techs. It was very difficult. The knowledge is improving …There are more residents and doctors. You can have a case and get help. In the past you couldn't consult. Now the mortality rate is reduced. We were very stressed in the past.” (Rwandan technician). “It is getting better. I come from another period where it was difficult to conduct anesthesia in Rwanda. Now we have more staff as specialists and technicians.” (Rwandan anaesthetist). “(Another) improvement is the residency program. We have good results because now we receive professors from different universities, especially from Canada and the USA… The anesthesia practice is improving.” (Rwandan anaesthetist). “The improvement I've seen is the recognition by the residents… they truly appreciate what's going on, see the big picture.” (American anaesthetist). Rwandan participants consistently expressed a desire for improvement and optimism about the future. “That sense of hope comes from not only what I see here in the hospital but also a swift move that I see in the entire country. I don't expect that anaesthesia will be left behind.” (Rwandan technician).

## Discussion

This study contributes knowledge of the practice of ANTS in a low-income setting; we found the ANTS framework to be highly relevant in this context. The common thread connecting the ANTS domains of task management, team working, situation awareness, and decision-making is communication. Communication in Rwandan anaesthesia providers is influenced by cultural patterns and resource limitations. Cultural elements include formality and hierarchy, lack of assertiveness and discomfort with taking leadership; these factors hinder discussion of plans and voicing of safety concerns. Resource shortages limit the ability of anaesthesia providers to practice safely. Anaesthesia providers are both frustrated by and resigned to poor outcomes. Communication in turn influences the practice of ANTS.

Future teaching interventions should be directed at improving communication skills, vital to safe anesthesia. Specifically, anaesthesia providers need practice in role definition, leadership, team planning and vigilance. Members of the entire operating theatre team – nursing, anaesthesia and surgery – should train together. This will help build a culture where anaesthesia providers, and other operating theatre personnel, feel empowered to speak up about safety concerns. This qualitative study will be followed by a quantitative evaluation to see whether ANTS in Rwanda improve with an educational intervention directed at communication skills. Future studies to explore team dynamics, including anaesthetists, surgeons and nurses, may further clarify the understanding of non-technical skills in Rwanda.

There were several strengths to this study. Our research team included anaesthetists with diverse perspectives: trainee and consultant, Rwandan and Canadian. Beginning with North American interviews allowed Rwandan anaesthesia providers to clarify misunderstandings. For example, some North American interview participants suggested that resignation to working in a resource-strained environment led to acceptance of poor outcomes by Rwandan anaesthesia providers. Later interviews in Rwanda clarified that, in fact, poor outcomes are a great source of frustration and anaesthesia providers are discouraged by the lack of capacity for better care. Data sources, including interviews, observations and field notes, were congruent, thereby adding validity to the results. By using only Rwandan investigators to observe study participants, the usual environment was maintained. We believe this minimized behaviour changes that might have resulted from being observed. All participants who were invited for interview gave consent. (This may reflect an inherent power dynamic whereby participants felt obligated; alternatively, this may reflect a strong desire among anaesthesia providers in Rwanda to improve their situation.) There were limitations in this study. ANTS were examined in academic, tertiary care centres only; practice in district hospitals in Rwanda may be different. We concentrated on anaesthesia personnel, while all members of the operating room team affect the practice of non-technical skills. Another limitation to the study was that although senior anaesthetists agreed to be interviewed, all but one declined to be observed by junior staff.

## Conclusion

Anaesthesia providers in Rwanda expressed frustration at their circumstances and desire for better patient outcomes. They recognized practice improvements due to morning team meetings, better education and an increased number of consultant anaesthetists. Future teaching interventions should address leadership and communication skills through encouraging both role definition and speaking up for patient safety. As described by this ethnographic study, the ANTS framework is highly relevant in both understanding practice and planning educational initiatives in resource-strained, low-income settings.
